# Effects of Maternal Pregnancy Intention, Depressive Symptoms and Social Support on Risk of Low Birth Weight: A Prospective Study from Southwestern Ethiopia

**DOI:** 10.1371/journal.pone.0096304

**Published:** 2014-05-21

**Authors:** Yohannes Dibaba Wado, Mesganaw Fantahun Afework, Michelle J. Hindin

**Affiliations:** 1 Department of Population & Family Health, College of Public Health and Medical Sciences, Jimma University, Jimma, Ethiopia; 2 School of Public Health, College of Health Sciences, Addis Ababa University, Addis Ababa, Ethiopia; 3 Johns Hopkins Bloomberg School of Public Health, John Hopkins University, Baltimore, Maryland, United States of America; Aga Khan University, Pakistan

## Abstract

**Background:**

Low birth weight (LBW) is the principal risk factor for neonatal and infant mortality in developing countries. This study examines the effects of unwanted pregnancy, prenatal depression and social support on the risk of low birth weight in rural southwestern Ethiopia. We hypothesized that unwanted pregnancy and prenatal depression increase the risk of low birth weight, while social support mediates this association.

**Methods:**

Data for the study comes from a prospective study in which women were followed from pregnancy through to delivery. Six hundred twenty two women were followed and 537 birth weights were measured within 72 hours. Multivariable log binomial regression was used to model the risk of low birth weight.

**Results:**

The mean birth weight was 2989 grams (SD±504 grams), and the incidence of LBW was 17.88%. The mean birth weight of babies after unwanted pregnancy was 114 g lower compared to births from intended pregnancy. Similarly, mean birth weight for babies among women with symptoms of antenatal depression was 116 grams lower. Results of unadjusted log-binomial regression showed that unwanted pregnancy, prenatal depression and social support were associated with LBW. The relationship between antenatal depressive symptoms and LBW was mediated by the presence of social support, while the association between LBW and unwanted pregnancy remained after multivariable adjustment.

**Conclusion:**

The incidence of low birth weight is high in the study area. Poverty, nonuse of antenatal care, low social support and unwanted pregnancy contribute to this high incidence of low birth weight. Hence, identifying women’s pregnancy intention during antenatal care visits, and providing appropriate counseling and social support will help improve birth outcomes.

## Introduction

Low Birth Weight (LBW), defined as birth weight under 2500 grams by the World Health Organization [1], is the principal risk factor for neonatal and infant mortality in developing countries. Studies show that LBW babies are more likely to die than heavier babies [2], [3], and nearly 60–80% of neonatal deaths occur among LBW infants [4]. Moreover, LBW is related to childhood motor skills, cognitive and social development [5]–[7]. The reduction of LBW is a key indicator for the attainment of lowering child mortality by two-thirds as part of the Millennium Development Goals (MDGs) [8]. The knowledge of the magnitude and determinants of LBW is important in developing policies aimed at reducing neonatal and infant mortality in countries with high neonatal mortality rates.

According to the United Nations Children’s Fund (UNICEF) and WHO, it is estimated that more than 20 million infants worldwide, representing 15.5% of all births, were LBW in 2000 [8]. The incidence of LBW in developing countries is more than double the level in developed regions (16.5% and 7% respectively), and over 95% of all LBW occurs in developing countries. Among developing countries, higher incidence of LBW has been reported from south Asian countries of India and Bangladesh [9]–[11]. Studies from Ethiopia indicate incidence rates ranging from 10–28% [12]–[17].

Studies have identified several risk factors for LBW, although the risk factors may differ between populations. In several studies, maternal height and pre-pregnancy weight, gestational weight gain, mid-upper arm circumference (MUAC), cigarette smoking, alcohol consumption and low socio-economic status were found to be the main risk factors for LBW [9], [15]–[19]. Socio-demographic factors such as infant’s sex, maternal age, primparity, maternal education and birth interval were also mentioned in several studies [15], [18]–[20]. Studies from Ethiopia reported that poverty, smaller MUAC size, lack of ANC attendance, infant sex, primparity and short maternal stature are associated with low birth weight [12]–[16]. However, very few studies have considered the effects of psychosocial factors such as pregnancy wantedness and prenatal depression on LBW in developing countries.

Unintended pregnancy may influence birth outcomes through increased levels of stress, and adoption of risky behaviors such as smoking, delayed initiation or inadequate prenatal care and reduced willingness to seek social support during pregnancy [21]–[23]. However, findings on the association between pregnancy intention and birth weight has been inconsistent. Several studies from developed countries found that pregnancy intention is not associated with birth weight [22]–[24], although some others reported a statistically significant association between unintended pregnancy and birth weight [25]–[27]. In a meta-analysis of observational studies from developed countries, however, Shah and colleagues observed an increased risk of LBW and pre-term birth among unintended pregnancies compared with intended pregnancies [21].

Some research suggests that depression during pregnancy can impair intrauterine growth and hence results in LBW [28], [29]. Several studies have shown that antenatal depression is associated with behaviors that are not conducive to a healthy pregnancy, including reduced attendance in antenatal care, increased substance use, and lower weight gain in pregnancy [22], [29], [30], which, in turn, increase the likelihood of LBW. However, empirical evidence supporting the association of antenatal depression with LBW has been equivocal. In developed countries, the majority of the studies reported no association between antenatal depression and LBW, except for few studies that disaggregated for socio-economic status and or race [31],[32]. In contrast, a number of studies from developing countries reported statistically significant associations between antenatal depression and LBW. Studies from south Asian countries of India, Pakistan and Vietnam and another study from Brazil reported that antenatal depression and other mental disorders were associated with LBW [33]–[36].

In contrast to depression and unwanted pregnancy, social support plays a buffering role from stressful life events by providing resources, support and strength during pregnancy [37]–[42]. In this study, we investigated whether women who reported unintended pregnancy or who experienced antenatal depression are at greater risk of having LBW infants. We hypothesized that the relationship between unintended pregnancy, depressive symptoms and LBW is mediated by social support.

## Methods and Materials

### Study Setting

The study was conducted in a Health and Demographic Surveillance System (HDSS) in Southwestern Ethiopia, which is located at about 260 km southwest of Addis Ababa (the capital). Data was collected from 11 HDSS kebeles (villages) consisting of more than 10,000 households and an estimated population of over 55,000. The area is predominantly rural and the population relies primarily on subsistence farming. Details of the study setting and sample are provided elsewhere [37].

### Study Design and Data Collection

The data for this study come from a community-based prospective cohort in which pregnant women were identified and followed to determine the effects of unintended pregnancy and prenatal depression on birth weight. All pregnant women in their 2^nd^ and 3^rd^ trimester living in the eleven kebeles (villages) from the HDSS were included in the study and were followed from pregnancy through delivery. Pregnant women were classified based on their pregnancy intention and prenatal depression. Data collection was done from June 2012 to February 2013. Six hundred twenty seven pregnant women were identified from the HDSS registration and from the records of Health Extension Workers who work in each village in June 2012. A baseline survey was conducted from June to July 2012 on 622 pregnant women. Ten female data collectors, with diploma level training and additional experience, collected the baseline data.

A structured questionnaire was administered to all study participants during the baseline survey.

The questionnaire was first developed in English and then translated to Oromo – a local language spoken in the study area, and back translated to English. As described elsewhere [37] translation and back translation of the depression scale was checked by psychiatrist. The EPDS has been validated in many developing countries including Ethiopia and is widely used for measuring antenatal depression. The questionnaire was piloted before use in a setting different from the study area.

### Ethical Considerations

The study was reviewed and approved by the Institutional review board of the college of Health Sciences, Addis Ababa University. Informed oral consent was obtained from every respondent. To this end, data collectors explained an information sheet prepared in the participant’s local language and asked for the consent of the participant. Interview was conducted with those who consented. A waiver of written consent was made by the IRB given that the data collection process does not involve more than minimal risk to the participants – there was no specimen/blood sample collection – and that the majority of the study participants can not read and write. All information gathered was anonymous to protect confidentiality. As described else where [37], all study participants were interviewed at their home in private area to ensure their privacy. In cases of illness including feelings of depression women were advised to consult health providers in the nearest health facility.

### Measurements

Birth weight was measured within 72 hours of delivery using Seca scale with accuracy within 100 grams. The scale was checked and calibrated before weighing. Birth weight was measured by data collectors who were based in each village. They were informed by village informants soon after the women delivered.

Data on pregnancy intention, depressive symptoms, social support during pregnancy and other explanatory variables were gathered using an interviewer-administered structured questionnaire during the baseline survey. All pregnant women were asked about their pregnancy intention, to recall their feelings at the time they became pregnant: “At the time you became pregnant, did you want to become pregnant then, did you want to wait until later, or did you not want to have any (more) children at all?” The responses were categorized as (1) wanted then “intended” (2) wanted later “mistimed” and not wanted at all “unwanted”. Moreover, questions related to happiness with pregnancy, husband’s perceived pregnancy intention were also asked of all women.

Depressive symptoms during pregnancy were measured using the Edinburgh Postnatal Depression Scale (EPDS). The items were scored on a scale of 0–3, allowing a total score ranging from 0 to 30. The internal consistency of the EPDS was tested using Cronbach alpha and was found to be 0.85. We used a cut of point of 13 and above on the scale to identify women with depressive symptoms. Social support was measured using the Maternity Social Support Scale (MSSS). The scale contains six items; each item measured on a five-point Likert scale and a total score of 30. The internal consistency of the scale was tested using Cronbach’s alpha and was found to be 0.74. We classified social support in to two categories based on the mean of the total score; high social support (for scores 24–30), and low social support (scores below 23). Details of the description of depression and social support measurement are presented elsewhere [37].

Mid Upper Arm Circumference (MUAC) measurements were made during the baseline survey using MUAC tape and following standard procedures. MUAC was measured during pregnancy as a proxy for maternal nutritional status. It was measured to the nearest 0.1 cm following standard procedures. A cut off point of 230 cm was used to show poor nutritional status. We also included several other explanatory variables based on previous studies including age (coded as <20, 20–29 and above 30 years), women’s education (none, primary and secondary and above), residence (rural or urban), wealth index, parity, sex of the newborn, history of stillbirth and antenatal care attendance during pregnancy. The wealth index was computed from ownership of the following household assets: radio, television, electricity, toilet, farm land, and number of animals such as cattle, sheep, and goats. As described elsewhere [37], a Principal Component Analysis (PCA) was conducted to compute wealth index and three principal components with eigenvalues greater than one were summed to obtain wealth index values. The resulting index was divided into three categories representing poor, middle and wealthy. Women were asked whether they have experienced a stillbirth in their lifetime to measure any experience of pregnancy loss. Moreover, women were asked whether they attended antenatal care for the index pregnancy.

### Data Analysis

Data were analyzed using STATA Version 11. First, bivariate analysis was done to compare birth weight by study characteristics using cross-tabulation and log binomial regression. Variables were included in to the multivariate model based on literature review and their association with LBW in the bivariate analysis. Multivariable log binomial regression was done to model the risk of LBW as function of demographic and psychosocial factors. Unadjusted and adjusted relative risks and 95% confidence intervals were reported. We assessed the potential of social support as a mediator using recommended procedures [43]. We checked for interaction between pregnancy intention and antenatal depression in predicting LBW. Moreover, multicollinearity between the variables was checked using variance inflation factor (VIF).

## Results

**Figure 1:**
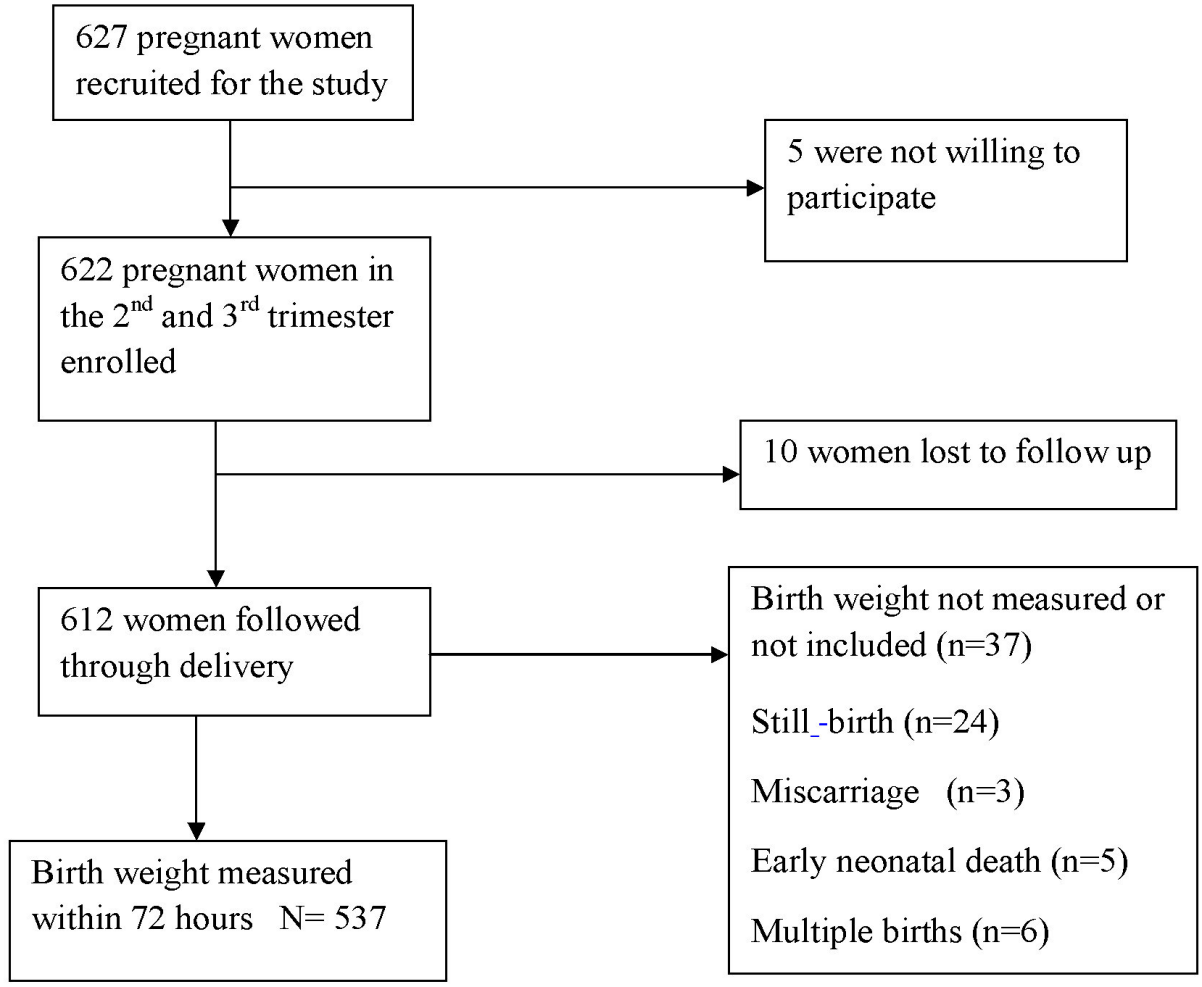
Flowchart showing the cohort and birth weight measurement, Southwest Ethiopia, 2012.

The characteristics of the study population at baseline are described elsewhere [37]. Majority of the study participants were from rural areas (76%), had no formal education (72%) and had an average parity of 4. About 41% of women reported that their pregnancy was not intended, while the magnitude of depressive symptoms in pregnancy was 20%. Of the 622 pregnant women enrolled in the study, birth weights of 537 births were included in the final analysis. The remaining 85 were not included because of failure to take weights within 72 hours (n = 37 births), occurrence of still births (n = 21), loss-to-follow up (n = 10), occurrence of multiple births (n = 6), early neonatal death (n = 5), and miscarriage (n = 3) (Figure 1). The mothers of included and excluded births were not different in terms of key socio-demographic variables including education, wealth index, MUAC, pregnancy intention, prenatal depression and parity (data not shown).

The mean birth weight was 2989 grams (SD±504 grams). Among the 537 neonates whose birth weight was taken, the incidence of LBW was 17.9%, (95% CI; 14.6, 21.1). A higher proportion of low birth weight neonates were born to women aged 35 and above (26%), rural women (20.3%), women in lowest wealth tertile (25.4%), high parity (20.3%), unwanted pregnancy (28.4%), small MUAC size (27.4%), antenatal depressive symptoms (26.2%), and low social support (22.4%) (Table 1). The mean birth weight of babies for women with an unwanted pregnancy was 114 grams lower than that of births from intended pregnancy. Similarly, mean birth weight of babies for women with symptoms of antenatal depression was 116 grams lower compared to birth of women who had no symptoms of depression during pregnancy (data not shown).

**Table 1:** Low birth weight by socio-demographic factors, pregnancy intention and antenatal depression, southwest Ethiopia, 2013.

Variables	N	Birth weight		Unadjusted OR, 95% CI	P
		>2500 g	<2500 g		
**Age**					
15–19	39	79.5	20.5	1.00	
20–29	337	83.7	16.3	0.76(0.33–1.73)	0.508
30–34	119	81.5	18.5	0.88(0.36–2.17)	0.780
35+	42	73.8	26.2	1.38(0.49–3.89)	0.548
**Sex of the newborn**					
Male	268	82.8	17.2	1.00	
Female	269	81.4	18.6	1.10(0.71–1.71)	0.67
**Educational status**					
No formal education	383	80.4	19.6	1.00	
Primary	128	85.2	14.8	0.72 (0.41–1.24)	0.232
Secondary & above	26	92.3	7.7	0.34(0.079–1.48)	0.151
**Residence**					
Rural	433	79.7	20.3	1.00	
Urban	104	92.3	7.7	0.33(0.15–0.70)	0.03
**Wealth tertile**					
Poor	181	74.6	25.4	1.00	
Middle	182	84.9	15.1	0.52 (0.31–0.89)*	0.017
rich	184	87.0	13.0	0.44(0.26–0.76)*	0.003
**Parity**					
0	80	83.8	16.3	1.00	
1–2	160	82.5	17.5	1.09(0.53–2.25)	0.808
3–4	169	82.8	17.2	1.07(0.52–2.18)	0.858
5+	128	79.7	20.3	1.31(0.63–2.74)	0.466
**Pregnancy Intention**					
Wanted then	314	85.4	14.6	1.00	
Mistimed	156	80.1	19.9	1.44(0.87–2.39)	0.151
unwanted	67	71.6	28.4	2.31(1.25–4.27)*	0.008
History of stillbirths					
Yes	51	76.5	23.5	1.00	
no	486	82.7	17.3	0.68(0.34–1.35)	0.27
**ANC use during pregnancy**					
Yes	295	86.8	13.2	1.00	
no	242	76.5	23.5	2.02(1.29–3.17)*	0.002
**Social support**					
Low	255	77.7	22.4	1.00	
High	282	86.2	13.8	0.56(0.36–0.87)**	0.01
**Depression during pregnancy**					
No	430	84.2	15.8	1.00	
Yes	107	73.8	26.2	1.89(1.14–3.12)*	0.01
**Mother’s MUAC**					
<230	113	72.6	27.4	1.00	
>230	424	84.7	15.3	0.48(0.29–0.78)*	0.003
Total	537	82.1	17.9		

Results of the unadjusted log binomial regressions shows that among socio-demographic and psychosocial factors; place of residence, wealth status, pregnancy intention, antenatal care use, social support, antenatal depressive symptoms and maternal MUAC were associated with low birth weight (Table 1). In a multivariate model with the three explanatory variables (Pregnancy intention, depression and social support) and after adjusting for socio-demographic factors, unwanted pregnancy was only marginally associated with LBW while the association of antenatal depression with LBW was attenuated. We examined whether the association between pregnancy intention, depressive symptoms and LBW was mediated by the presence of social support and run separate multivariate models with unintended pregnancy and depression as key independent variables. Multivariate Models I and II show that unintended pregnancy is significantly associated with LBW after adjusting for social support and other socio-demographic variables (Table 2). Similarly, multivariate models III and IV show that depression in pregnancy is associated with LBW after adjusting for social support and other socio-demographic variables, although the relative risk is lowered. Social support was significantly associated with LBW. The risk of giving birth to LBW was lower for women who had better social support during pregnancy compared to those who had lower social support.

**Table 2:** Relative risks from log binomial regression analysis showing association between pregnancy intentions, maternal depression and social support with Low Birth Weight, Southwest Ethiopia, 2013.

Variables	Model IRR, (95% CI)	Model IIRR, (95% CI)	Model IIIRR, (95% CI)	Model IVRR, (95% CI)
**Pregnancy intention**				
Intended (reference)	1.00	1.00		
Mistimed	1.24(0.72–2.12)	1.25(0.73–2.14)		
Unwanted	2.12(1.05–4.28)*	2.08(1.02–4.23)*		
Depression in pregnancy			1.87(1.09–3.21)*	1.77(1.03–3.04)*
High Social support		0.59(0.37–0.94)*		0.59(0.36–0.94)*
**Maternal age**				
15–19	1.00	1.00	1.00	1.00
20–29	0.59(0.22–1.63)	0.57(0.21–1.59)	0.56(0.23–1.37)	0.53(0.21–1.31)
30–34	0.65(0.20–2.13)	0.62(0.18–2.03)	0.59(0.22–1.65)	0.56(0.20–1.56)
35+	0.86(0.22–3.28)	0.71(0.18–2.80)	0.88(0.28–2.81)	0.75(0.23–2.41)
**Mother’s education**				
No education	1.00	1.00	1.00	1.00
Primary	0.82 (0.45–1.49)	0.76(0.41–1.41)	0.90(0.49–1.67)	0.86(0.47–1.59)
Secondary & above	0.48(0.10–2.21)	0.45(0.10–2.16)	0.51(0.11–2.42)	0.50(0.11–2.40)
Urban Residence	0.49(0.22–1.13)	0.46(0.20–1.05)	0.45(0.20–1.04)	0.43(0.19–0.98)
**Wealth tertile**				
Lower (reference)	1.00	1.00	1.00	1.00
Middle	0.52 (0.31–0.89)*	0.54(0.31–0.96)*	0.54(0.31–0.96)*	0.57(0.31–1.01)
Upper	0.44(0.26–0.76)*	0.50(0.27–0.90)*	0.53(0.30–0.93)*	0.55(0.31–0.97)*
No ANC received	2.02(1.29–3.17)*	1.73(1.08–2.78)*	1.65(1.03–2.64)*	1.66(1.04–2.67)*
Has history of still births	0.68(0.34–1.35)	0.72(0.34–1.53)	0.69 (0.32–1.46)	0.72(0.34–1.54)
**Maternal MUAC**				
<230 cm (Reference)	1.00	1.00	1.00	1.00
>230 cm	0.48(0.29–0.78)*	0.59(0.37–0.94)*	0.56(0.33–0.93)*	0.56(0.36–0.94)*

*p<0.05;

**p<0.01;

***P<0.001.

The other factors that were significantly associated with LBW in the final model were wealth status, ANC use and maternal MUAC size. LBW was significantly lower among births from middle and upper wealth status women compared with women of lowest wealth status. The risk of giving birth to LBW was significantly higher for women who did not use ANC during pregnancy compared to those who used ANC. Moreover, the risk of giving birth to LBW was about 40% lower for women who had higher MUAC size (Table 2).

## Discussion

This study examined the associations between psychosocial factors (pregnancy intention, maternal antenatal depression and social support) and LBW in Ethiopia. Our findings show that LBW is common in the study area, with the incidence of 17.9% (95% CI; 14.6, 21.1). In Ethiopia, nearly nine in ten babies are delivered at home and are not weighted at birth. In particular, birth weight is not known for rural babies and such community-based studies focusing on rural populations find higher incidence of LBW than facility-based studies [13], [14]. For instance, recent community based studies in eastern and southern Ethiopia found higher incidence of LBW of 28% and 17% respectively [15], [16]. In the 2011 Ethiopian Demographic and Health Survey, a nationally representative population-based sample, reported that 21% of all live births in rural Ethiopia in the five years preceding the survey were very small in size, as reported subjectively by the mother [17].

Our regression analysis showed that unwanted pregnancy and depressive symptoms were associated with LBW at the bivariate level, and in separate models not including any of the two variables. The association between LBW and unwanted pregnancy remained marginally significant after multivariable adjustment. Accordingly, births after unwanted pregnancy but not a mistimed pregnancy were more likely to be LBW. Unwanted pregnancy could contribute to LBW through adoption of risky behaviors such as non use or inadequate prenatal care, increased stress and depression and reduced social support during pregnancy. Several previous studies reported inconsistent relationships between pregnancy intention and LBW [22]–[24], although a meta-analysis of observational studies found an increased risk of LBW among births after unwanted and mistimed pregnancies [21]. However, the relationship between antenatal depressive symptoms and LBW was attenuated in the multivariate models adjusting for pregnancy intention, other social demographic factors and social support indicating that the relationship is partly mediated by the presence of social support. The association between depression and LBW has not been consistent although a meta-analysis has shown that prenatal depression increases the risk of LBW and pre-term birth [39]. A previous study from Ethiopia also showed that antenatal common mental disorders such as stress and depression are not associated with low birth weight and pre-term births in Butajira, Central Ethiopia [44].

We thought to understand if the association between unwanted pregnancy, depressive symptoms and LBW was mediated by the presence of social support, and found that social support plays a mediating role between depressive symptoms and LBW but not between pregnancy intention and LBW. We also tested for interactions and found significant interactions between unwanted pregnancy and antenatal depression in predicting LBW. As shown else where [37], unwanted pregnancy is significantly associated with antenatal depression in the baseline study and that might have affected the strength of the relationship between depression and LBW in addition to the mediating influence of social support. There were several studies from developing countries that reported significant associations between antenatal depression and LBW [33]–[36], however, many of these studies did not adjust for the effects of other psychosocial factors including social support and pregnancy wantedness.

Social support, which measured the presence of support from families, friends and the partner during pregnancy, was significantly associated with LBW. The risk of LBW was significantly lower among women with higher social support during pregnancy. Several studies from developed countries have reported the role of social support in better birth outcomes [40], [41]. It has been hypothesized that social support has a mediating effect on the relationship between life stress and poor pregnancy outcomes such as LBW. It plays a buffering role from stressful life events by providing resources, support and strength during pregnancy [42].

Other factors independently associated with LBW in the multivariate analysis included wealth status, use of antenatal care and maternal MUAC. The effect of wealth status on LBW has been observed in several studies [9], [16], [18], [19]. These studies have shown that births from poor households were more likely to be of LBW than those from better off families. This might be related to the sort of maternal nutrition and pregnancy care obtained under higher SES situations. Lack of antenatal care was also associated with the risk of LBW. Several previous studies reported on the role of ANC in improved birth outcome [16], [45], [46]. Antenatal care provides a series of medical, nutritional and education interventions to reduce the incidence of LBW and adverse pregnancy conditions. In this study, 47% of women did not attend any antenatal care during pregnancy. As a result they did not obtain education and counseling on proper nutrition and balanced diet as well as other routine checkups for weight, height, gestational weight gain, hypertension and related health conditions that are given during ANC visits in Ethiopian health facilities. These interventions could make the differences with women who attended antenatal care. Maternal MUAC size, a proxy for nutritional status was also associated with LBW. Several studies have reported similar findings [15.16, 47,48]. MUAC is found to be a good anthropometric indicator to identify acutely malnourished pregnant women and to predict adverse birth outcomes [49]. The association shows that maternal nutritional status is one of the factors that directly influence birth weight, as better maternal nutrition results in higher birth weight.

## Conclusion

LBW is common in the study area. Nearly 18% of births to women who were followed from pregnancy through delivery had LBW, indicating that LBW is still an important health problem in rural Ethiopia. The incidence of LBW was higher among women whose pregnancy was unwanted and among those with antenatal depressive symptoms. The relationship between antenatal depressive symptoms and LBW was mediated by the presence of social support, while the association between LBW and unwanted pregnancy remained after multivariable adjustment. The presence of social support was protective, buffering the effects of depression on birth outcomes. Other important factors that significantly influenced birth weight were wealth status, use of antenatal care in pregnancy and maternal MUAC size. Hence, identifying women’s pregnancy intention during antenatal care visits, encouraging use of antenatal care during pregnancy and providing appropriate counseling and social support will help improve birth outcomes.
